# Supplementary material for: Preparation of Electrospun Active Molecules Membrane Application to Atmospheric Free Radicals
Source: Membranes (Basel). 2022 Apr 29;12(5):480. doi: 10.3390/membranes12050480 (PMC9143268; doi:10.3390/membranes12050480)
Supplement: Supplementary file 1 [file membranes-12-00480-s001.zip › membranes-1685685-supplementary.pdf]

Table S1: Electrospinning parameter settings

**Number of polymers added(g)**	**Spinning voltage (kV)**	**Receiving distance (cm)**	**Push injection speed (mm/min)**
0.1300	10.00	13.00	0.130
0.1500	12.00	15.00	0.155
0.1700	14.00	17.00	0.180
0.1900	16.00	19.00	0.205
0.2100	18.00	21.00	0.230

Table S2: Experimental results of fluorescence detection precision

	Chrysin	Baicalein	Scutellarein	Genistein	Quercetin	Baicalin
OH	3.50	0.22	0.17	2.06	0.35	0.20
O_3	0.80	0.27	0.53	0.08	0.12	0.18
ROS	1.01	0.76	0.11	0.13	0.49	0.25

Table S3: Experimental results of fluorescence detection stability

	Chrysin	Baicalein	Scutellarein	Genistein	Quercetin	Baicalin
OH	0.09	0.26	0.42	0.23	0.22	0.15
O_3	0.12	0.37	0.17	0.54	0.40	0.29
ROS	0.19	0.25	0.90	0.29	0.39	0.11

Table S4: Pollutant averages for the second quarter of 2021

	PM_{10} $\mu g/m^3$	$PM_{2.5}$ $\mu g/m^3$	SO_2 $\mu g/m^3$	NO_2 $\mu g/m^3$	CO mg/m^3	O_3 $\mu g/m^3$	TVOC $\mu g/m^3$
Average value	78.56	35.19	15.93	40.59	0.67	78.29	462.03

Table S5: The data of AQI etc. at the same stage of sampling for the detection of free radicals

	Sample Number	T ℃	Humidity %	AQI	$PM_{2.5}$ $\mu g/m^3$	PM_{10} $\mu g/m^3$	SO_2 $\mu g/m^3$	NO_2 $\mu g/m^3$	O_3 $\mu g/m^3$	CO mg/m^3	Free Radicals FL. $10^8/cm^3$
	504(09:00-12:00)	13.4	25	44	17	44	6	41	62	0.5	3.76±0.09
	504(10:00-13:00)	15.4	50	55	31	59	22	55	18	1.4	5.18±0.25
Chrysin	504(11:00-14:00)	20.7	43	78	15	106	16	80	10	1.0	4.11±0.01
	504(12:00-15:00)	22.7	23	41	18	41	5	26	120	0.2	3.83±0.07
	526(12:00-15:00)	26.4	16	44	19	44	9	39	126	0.3	3.78±0.08
	423(09:00-12:00)	18.8	25	44	17	44	6	41	62	0.5	3.65±0.15
	424(11:00-14:00)	16.7	38	67	31	83	10	117	60	1.0	5.87±0.37
Baicalein	423(11:00-14:00)	16.7	41	56	36	61	26	47	42	1.2	4.16±0.03
	508(10:00-13:00)	23.8	21	41	18	41	5	26	120	0.2	3.58±0.16
	526(09:00-12:00)	27.6	13	45	13	36	6	19	145	0.2	4.14±0.03
	505(09:00-12:00)	24.5	32	51	32	52	17	56	104	0.8	4.57±0.24
	505(11:00-14:00)	26.4	45	67	31	83	10	117	60	1.0	5.18±0.40
Scutellarein	505(12:00-15:00)	25.4	38	69	22	88	16	83	37	0.7	3.95±0.47
	522(12:00-15:00)	29.8	39	39	10	39	9	23	102	0.4	3.90±0.06
	522(12:00-15:00)	17.9	34	72	33	93	32	68	44	0.7	4.61±0.02
	502(11:00-14:00)	31.2	45	67	31	83	10	117	60	1.0	7.16±0.26
	427(13:00-16:00)	27.4	37	59	34	67	12	54	87	0.5	6.17±0.09
Genistein	522(13:00-16:00)	30	35	38	14	38	9	23	93	0.4	6.25±0.10
	522(13:00-16:00)	22.4	28	62	31	73	16	46	88	0.5	5.24±0.43
	522(14:00-17:00)	26.3	26	47	30	47	7	24	114	0.3	6.87±0.21
Quercetin	503(15:00-18:00)	26.5	23	35	7	30	10	37	111	0.4	3.71±0.38

	Sample Number	T ℃	Humidity %	AQI	$PM_{2.5}$ μg/m³	PM_{10} μg/m³	SO_2 μg/m³	NO_2 μg/m³	O_3 μg/m³	CO mg/m³	Free Radicals FL. $10^8/cm^3$
Baicalin	506(14:00-17:00)	24.6	27	58	36	66	11	60	123	0.6	4.46±0.02
	517(15:00-18:00)	21.2	34	73	27	96	26	93	74	0.7	5.06±0.16
	517(14:00-17:00)	19.9	34	47	21	47	14	32	77	0.6	5.50±0.26
	517(14:00-17:00)	26.3	26	47	30	47	7	24	114	0.3	4.09±0.06
	503(14:00-17:00)	29.8	22	45	12	45	6	13	118	0.2	3.79±0.23
	517(13:00-16:00)	27.8	27	39	19	32	5	28	124	0.3	3.68±0.15
	517(14:00-17:00)	21.9	35	62	37	74	12	73	65	0.6	4.00±0.29
	517(15:00-18:00)	26.3	33	36	7	36	9	24	108	0.3	4.10±0.10
	517(15:00-18:00)	22.6	27	48	24	48	10	28	104	0.3	3.68±0.11
